# Nonlinear Waveform Optimization for Enhanced Ink Droplet Formation in Material Jetting

**DOI:** 10.3390/mi16040445

**Published:** 2025-04-09

**Authors:** Qintao Shen, Li Zhang, Renquan Ji, Viboon Saetang, Huan Qi

**Affiliations:** 1 College of Mechanical Engineering, Zhejiang University of Technology, Hangzhou 310014, China; 2112002203@zjut.edu.cn (Q.S.);; 2 Advanced Materials Additive Manufacturing Innovation Research Center, Hangzhou City University, Hangzhou 310015, China; 3 Zhejiang-Thailand International Joint Laboratory on New Materials Digital Design and Processing Technology, Hangzhou City University, Hangzhou 310015, China; 4 School of Engineering, Hangzhou City University, Hangzhou 310015, China; 5 Department of Production Engineering, Faculty of Engineering, King Mongkut’s University of Technology Thonburi, Bangkok 10140, Thailand

**Keywords:** material jetting, droplet formation, numerical simulation, pressure fluctuation, waveform optimization, CNN, PSO

## Abstract

Material jetting, as a critical additive manufacturing technology, relies on precise control of the driving waveform to achieve high-quality droplet formation. During the droplet ejection process, pressure fluctuation at the nozzle outlet plays a significant role in droplet formation. Traditional experimental methods for optimizing the driving waveform often struggle to address the complex nonlinearities inherent in the jetting process. In this study, a numerical simulation model of the droplet ejection process is established to elucidate the influence mechanism of nozzle outlet pressure oscillations on droplet formation. A novel optimization method combining Convolutional Neural Networks (CNNs) and Particle Swarm Optimization (PSO) is proposed, targeting the suppression of residual pressure oscillations and achieving the desired pressure fluctuation. The method leverages nonlinear regression and optimization to obtain the optimal waveform design. Simulation and experimental results demonstrate that the optimized waveform effectively suppresses residual pressure oscillations, significantly improves droplet formation quality, and reduces pressure fluctuation convergence time by approximately 32.19%. The findings demonstrate that the optimized waveform effectively improves droplet ejection quality and stability.

## 1. Introduction

Additive manufacturing technology holds broad application prospects in fields such as electronics, healthcare, aerospace, and energy [1,2,3,4,5]. As a type of additive manufacturing, material jetting is renowned for its high precision, high flexibility, and excellent material utilization efficiency [6,7,8,9,10]. Based on the operational mode, material jetting can be classified into continuous inkjet printing and Drop-on-Demand (DoD) inkjet printing [11]. Among these, DoD inkjet printing is the most used material jetting technology. It generates droplets with higher positional accuracy and smaller droplet sizes compared to continuous inkjet printing by controlling piezoelectric or thermoelectric actuators to produce instantaneous pressure [12]. Since the DoD inkjet printing process involves the collaborative coupling of multiple physical fields such as thermal, mechanical, fluid, and electrical domains, effectively controlling the droplets and ensuring print quality and efficiency has become a critical technical challenge in this field.

Bogy et al. [13] experimentally demonstrated that the inkjet printing process is closely related to the propagation of pressure waves within the nozzle ink chamber, leading to the widely accepted wave conduction theory. Based on this theory, and assuming the nozzle structure remains unchanged, the inkjet print quality largely depends on the rheological properties of the ink and the driving voltage parameters of the ejection process, which has been extensively studied by many scholars. Fromm et al. [14], through a systematic analysis of inkjet mechanisms, introduced the Oh number, which is independent of ejection speed, to describe the behavior of the ink droplet. They also proposed a dimensionless constant Z > 2 to determine stable inkjet printing conditions. Reis and Derby et al. [15,16] extended Fromm’s criterion for stable inkjet printing to a range of 10 > Z > 1 through numerical simulations. When Z < 1, the ink viscosity is too high, preventing effective ink ejection from the nozzle, whereas when Z > 10, the inkjet printing process is accompanied by unnecessary satellite droplets. Richardot et al. [17] proposed a comprehensive evaluation method for the printability of viscoelastic inks by measuring the high-frequency viscoelastic properties of the ink, calculating its Deborah number (De), and constructing a De-We diagram in combination with the We. The optimal range for stable single-drop ejection was determined to be 0.1 < De < 1 and 2 < We < 15. The inkjet stability criterion provides a valuable reference for selecting appropriate printing conditions for DoD inkjet printing. However, this criterion primarily focuses on the rheological properties of the ink and the nozzle dimensions. It cannot fully assess droplet performance during actual printing, as the formation of ink droplets is also directly influenced by the nozzle drive waveform parameters [18,19].

Numerous scholars have conducted research on the influence of the driving waveform during the inkjet ejection process. Lyu et al. [20] introduced a novel odd harmonic method for the elimination of satellite droplets. Kim et al. [21] analyzed the piezoelectric inkjet process using both experimental and numerical methods and proposed a supply pressure control method to suppress residual pressure vibrations during the ejection process. This method was found to improve inkjet efficiency by approximately 51.5%. Cheng and Tseng et al. [22] conducted experimental studies on the impact of waveform parameters on the performance of multi-nozzle printheads and proposed a corresponding design method for multi-nozzle printhead driving waveforms. Yang et al. [23] designed nozzle-driving waveforms using the modulation of cross-talk effects inside and outside the printhead, achieving nearly a tenfold increase in droplet ejection speed and distance while significantly expanding the compatibility of printable ink materials. Wang et al. [24] derived a calculation formula for the jet volume flow rate corresponding to ink droplets produced by different fluid materials and used an iterative algorithm to automatically calculate the optimal driving waveform corresponding to the jet volume flow rate. With the rapid development of artificial intelligence algorithms in recent years, their application in optimizing inkjet print quality has gradually gained attention. Yun et al. [25] proposed a piezoelectric waveform optimization method based on a root growth algorithm to search for the driving waveform parameters that meet specific ink droplet characteristics. Segura et al. [26] established a network framework based on tensor time series analysis to predict droplet behavior in the inkjet process, which can accurately predict outcomes for known or unknown materials and process parameters. Brishty et al. [27] successfully predicted the influence of driving signals and ink properties on the ejection speed, radius, and other characteristics of the ink droplets using machine learning methods. Kim et al. [28] introduced a closed-loop machine learning process to recommend optimal driving waveforms for satellite-free inkjet printing. Huang et al. [29] proposed using deep recurrent neural networks for unsupervised learning of inkjet process videos to understand the formation and movement of ink droplets. Kim et al. [30] developed a machine learning-based model that integrates the De and Oh to predict the jetting behavior of viscoelastic inks. The model can even automatically optimize the driving waveform based on simple rheological measurements to achieve precise and efficient printhead control. In addition, techniques such as Random Forest [31,32], Support Vector Machines [32,33], Decision Trees [33], and Neural Networks [34,35] have also been applied in ink droplet ejection optimization.

Currently, the optimization of drive waveforms for DoD inkjet printing is primarily based on experimental methods. However, in addition to the cumbersome nature of the experimental process, many parameters cannot be accurately quantified. Furthermore, due to the inherent nonlinear behaviors in the ink droplet ejection process, the optimization is highly prone to local optima. CNN, as a type of deep feedforward neural network, is well known for its ability to automatically extract target features. By learning feature patterns from sample datasets, CNN effectively eliminates the inefficiencies and low classification accuracy associated with traditional manual feature extraction, making it particularly advantageous for handling complex nonlinear problems [36,37]. Meanwhile, PSO stands out among optimization algorithms due to its strong global search capability, fast convergence, simple parameter settings, and ease of implementation [38,39]. The integration of CNN and PSO effectively addresses the challenges in optimizing driving waveforms. Leveraging adaptive learning capabilities, CNN can establish a nonlinear mapping model between waveform parameters and ejection performance while PSO efficiently searches for the optimal waveform configuration. Together, they enable accurate prediction and real-time adjustment of the ejection performance, significantly enhancing the effectiveness of waveform optimization. This study develops a droplet ejection model under multi-field coupling to investigate the pressure fluctuations at the nozzle caused by driving waveforms during the ejection process. The relationship between droplet ejection behavior and pressure fluctuations is verified through experimental and simulation comparisons. Based on the simulation model, CNN regression and PSO algorithms are applied to optimize the driving waveform, suppressing residual pressure fluctuations at the nozzle during ejection. This optimization improves droplet ejection quality and stability.

## 2. Experimental Details

### 2.1. Ink Composition and Rheological Properties

**Figure 1:**
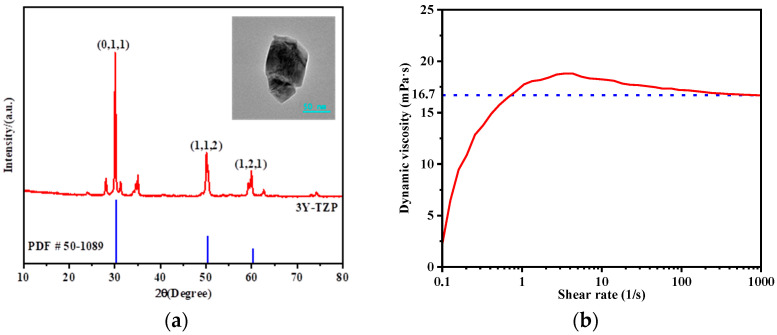
Ink properties: (**a**) XRD and TEM images of zirconia particles; (**b**) dynamic viscosity of the ink as a function of shear rate.

The ink used in this study is composed of 3 mol% yttria-stabilized tetragonal zirconia polycrystals (3Y-TZP) as the primary functional material, with an average particle size of approximately 100 nm. The X-ray diffraction (XRD) and transmission electron microscopy (TEM) images of the particles are shown in Figure 1a. The solid content of the ink ranges from 42 to approximately 47 wt%. Ethylene glycol is used as the solvent carrier, with a content of 53.0~58.0 wt%. Polyvinyl alcohol (PVA)-based polymers are employed as the primary dispersing agents, with the total content of PVA and other additives ranging from 1.0~3.0 wt%.

The density of the ink was measured using an electronic balance (BSM-420.3, Shanghai Zhuojing Electronic Technology Co., Ltd., Shanghai, China), and the rheological properties were tested using a rheometer (Mars40, Haake, Vreden, Germany). While high-frequency viscoelastic behavior and extensional rheology play critical roles in droplet dynamics characterization [40], the current study employs essential viscosity data under jetting-relevant shear conditions. The surface tension was measured with a surface tensiometer (Q1000, Shanghai Fangrui Instruments Co., Ltd., Shanghai, China). It should be noted that dynamic surface tension would better reflect the rapid interface evolution during ligament formation, though static equilibrium values are reported here for model simplification. All measurements were conducted at room temperature (25 °C) to ensure consistency with the jetting conditions. The ink’s density is determined to be 1470 kg/m^3^, and its dynamic viscosity, as a function of shear rate, is shown in Figure 1b. Since the ink is generally exposed to high shear rates during the jetting process [41], it can be approximated as a Newtonian fluid with a viscosity of 16.7 mPa·s and a surface tension of 39.7 mN/m.

### 2.2. Inkjet Observation System

**Figure 2:**
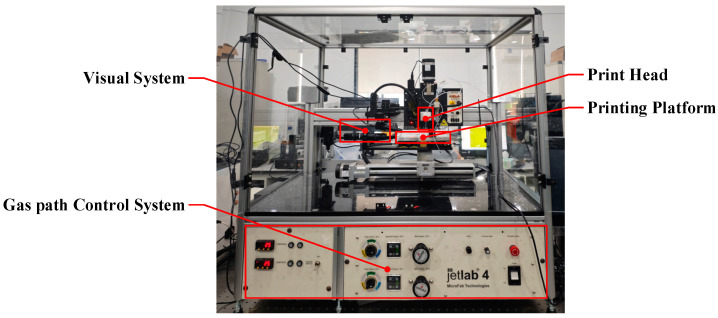
Inkjet observation system.

The inkjet observation system is used to experimentally validate the simulation results. The system is based on the Jetlab4 inkjet printing system from MicroFab, Plano, TX, USA, designed for nanomaterial deposition. Figure 2 shows a photo of the Experimental equipment. The visual system consists primarily of a high-speed CCD camera and a strobe light. With the assistance of a synchronized trigger, the high-speed CCD camera captures the inkjet process in real-time. The strobe light provides high-intensity, short-duration pulses of illumination, ensuring clear imaging by the high-speed CCD camera. The printhead used is the MicroFab MJ-AT-01-50 model (Plano, TX, USA), with a nozzle diameter of 50 microns, driven by an extrusion-type piezoelectric actuator. The actuator generates radial expansion and contraction in response to voltage polarity changes. To stabilize the initial liquid meniscus position at the nozzle during the inkjet process, a supply pressure of −2 kPa is applied to the ink supply end of the printhead.

### 2.3. Model Setup

To track the interface changes between the ink droplet and air, the level set method [42] is used to describe the two-phase interface. At the interface transition, the level set function ϕ smoothly transitions from 0 to 1, where ϕ=0 represents air, ϕ=0.5 represents the air–ink interface, and ϕ=1 represents the ink.

(1) $$\phi =\left\{\begin{array}{l} 0\leq \phi <0.5\;\;,\;\;\;Air\\ 0.5\;\;\;\;\;\;\;\;\;\;\;\;\;\;\;\;\;,\;\;\;Interface\\ 0.5<\phi \leq 1\;\;,\;\;\;Ink \end{array}\right.$$

To ensure the conservation of mass during the computation, the level set function is reinitialized after each time step. The reinitialized level set function for the advection transport equation can be expressed as

(2) $$\frac{\partial \phi }{\partial t}+u\cdot ▽\phi =\gamma ▽\cdot \left[\varepsilon \cdot ▽\phi -\phi \cdot \left(1-\phi \right)\cdot \frac{▽\phi }{\left|▽\phi \right|}\right]$$

In the equation, t represents time, u is the velocity, γ is the re-initialization parameter, typically set to the estimated maximum fluid velocity, and ε is the interface thickness control parameter, generally taken as half of the maximum grid size. The specific values of the re-initialization parameters and interface thickness control parameters can be found in Table A1 of Appendix A.1.

To prevent oscillations in the numerical computation process caused by abrupt changes in density and viscosity at the fluid interface, a smoothing transition is applied using the level set function.

(3) $$\rho \left(\phi \right)=\rho_{air}+\left(\rho_{ink}-\rho_{air}\right)\phi \;\mu \left(\phi \right)=\mu_{air}+\left(\mu_{ink}-\mu_{air}\right)\phi$$

In the equation, $\rho_{air}$ represents the air density, $\rho_{ink}$ represents the ink density, $\mu_{air}$ represents the air viscosity, and $\mu_{ink}$ represents the ink viscosity.

The inkjet ejection process is a typical laminar flow. It is assumed that the fluid is incompressible and that there is no temperature change during the ejection process. Based on fluid mechanics theory, the incompressible Navier–Stokes equations under the effects of surface tension and gravity are as follows:

(4) $$\rho \left(\frac{\partial u}{\partial t}+u\cdot ▽u\right)=▽\cdot \left(\mu \left(▽u+▽u^{T}\right)\right)-▽p+F_{st}+\rho g$$

(5) ρ▽⋅u=0

In the above equations, ρ represents the density, u is the velocity vector, t is the time, p is the pressure, $F_{st}$ is the surface tension, and g is the gravitational acceleration. Surface tension plays a crucial role in the inkjet ejection process. In this study, the continuous surface tension model proposed by Brackbill et al. [43] is used, and its expression is as follows:

(6) $$F_{st}=\sigma \delta \kappa \frac{▽\phi }{\left|▽\phi \right|}$$

In this formula, σ represents the surface tension coefficient, and δ is the Dirac delta function, which simplifies to $\delta =6\left|\phi \left(1-\phi \right)\right|\left|▽\phi \right|$. This function is defined as 1 only at the interface of the two-phase fluid, ensuring that surface tension acts solely at the interface. The curvature of the interface, κ, is given by $\kappa =-▽\cdot \frac{▽\phi }{\left|▽\phi \right|}$. This formulation ensures that surface tension is accurately modeled at the fluid interface, a critical aspect of the droplet ejection process.

**Figure 3:**
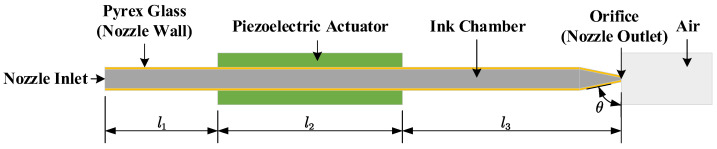
Geometric model of the ejection model.

**Figure A1:**
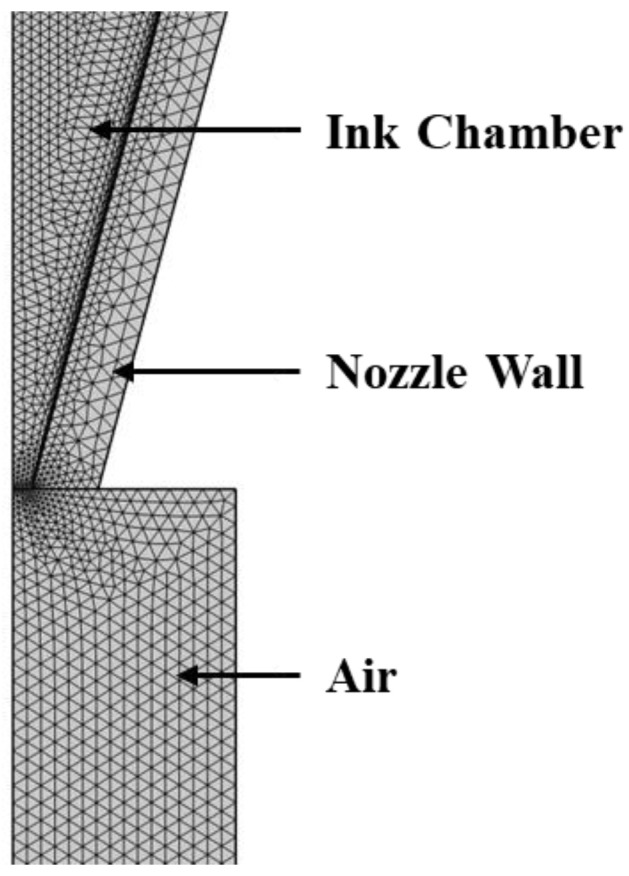
Mesh diagram at the nozzle.

The modeling and simulation were carried out using the commercial software COMSOL Multiphysics 6.2. Based on the physical design of the nozzle, a geometric model for the inkjet system was established, as shown in Figure 3. The entire model is divided into four main components: the ink chamber, nozzle wall, piezoelectric actuator, and air domain. The material for the nozzle wall is selected as Pyrex glass, and the piezoelectric material for the actuator is chosen as PZT-5H. The key dimensional parameters of the geometric model are provided in Table A2 of Appendix A.2. To improve computational efficiency, the model was simplified to a 2D axisymmetric configuration. The mesh was generated using free triangular elements with adaptive mesh refinement to dynamically refine the grid and improve the computational accuracy. The mesh images and mesh sensitivity analysis data can be found in Figure A1 and Table A3 of Appendix A.3.

**Figure 4:**
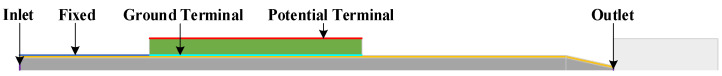
Boundary conditions for the ejection model.

The droplet ejection process from the nozzle involves piezoelectric effects, fluid–solid coupling, and multiphase flow. To ensure the rationality of the ejection model and the accuracy of the simulation results, appropriate boundary conditions need to be set. The boundary conditions for the ejection model are shown in Figure 4. At the nozzle inlet, which serves as the ink supply port, an inlet boundary condition is set with a reference static pressure of −2 kPa, as in the experimental setup, to counteract the effects of gravity and form a stable meniscus. The nozzle outlet is set with an outlet boundary condition, where the pressure is specified as atmospheric pressure. The inner surface of the piezoelectric actuator in contact with the nozzle’s outer wall is set to a grounded boundary condition. The outer surface of the piezoelectric actuator is set with a potential boundary condition, where the potential is specified according to the experimental drive voltage waveform to actuate the piezoelectric actuator. The outer wall of the nozzle at the inlet, which is fixed to the inkjet observation system, is set with a fixed constraint boundary condition. Additionally, a wetting wall boundary condition is applied to the nozzle walls, with a fixed contact angle of π/2 to ensure consistent meniscus formation and stability. For a detailed summary of the boundary conditions and solver settings used in the numerical model, please refer to Appendix A.4.

### 2.4. Drive Waveform

**Figure 5:**
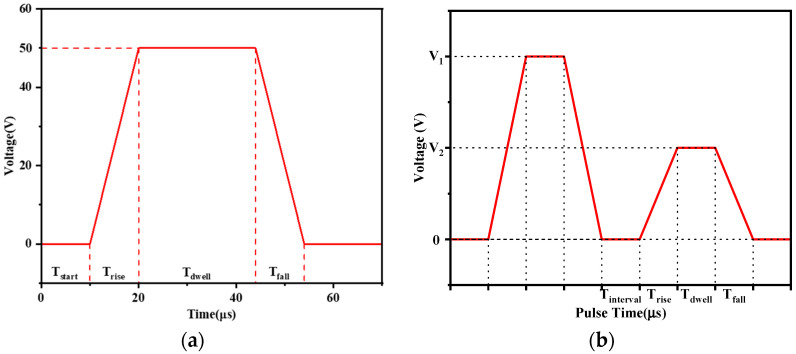
Drive waveform: (**a**) single waveform; (**b**) double waveform.

The drive waveform of the piezoelectric printhead can be categorized into single waveforms, double waveforms, and bipolar waveforms based on their shape [18]. The single waveform, as shown in Figure 5a, consists of a rise time $T_{rise}$, a dwell time $T_{dwell}$, and a fall time $T_{fall}$. During the rise time, the driving voltage increases, causing the piezoelectric actuator to deform and generate a pressure wave within the ink chamber. During the dwell time, the pressure wave propagates and reflects within the ink chamber until the fall time, when the driving voltage decreases, allowing the piezoelectric actuator to return to its original shape and generate a directional pressure wave that successfully ejects the ink droplet. According to pressure wave propagation theory, by selecting an appropriate dwell time, the nozzle ejection pressure can be maximized. In this study, the driving waveform used in both the experimental and simulation processes is a single waveform with a starting time $T_{start}$ of 10 μs, a rise time $T_{rise}$ of 10 μs, a dwell time $T_{dwell}$ of 24 μs, a fall time $T_{fall}$ of 10 μs, and a driving voltage $V_{1}$ of 50 V.

The double waveform, as shown in Figure 5b, is generated by superimposing two single waveforms in the same direction. By appropriately adjusting the parameters of the second single waveform, the residual oscillations from the first waveform can be quickly suppressed, thereby improving the ejection efficiency. In this study, the subsequent waveform optimization utilizes the double waveform.

## 3. Simulation Results and Discussion

### 3.1. Droplet Formation Process

**Figure 6:**
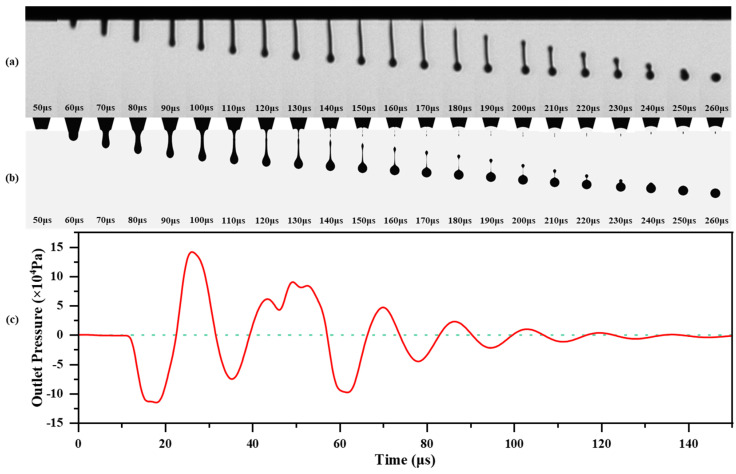
Ejection experiment and simulation results: (**a**) grayscale image of ink droplet ejection experiment; (**b**) ink droplet ejection simulation results; (**c**) simulation results of ink pressure fluctuation at the nozzle outlet.

Figure 6a shows the droplet formation process during the ejection experiment. The pressure wave generated during the voltage rise phase propagates and reflects within the ink chamber during the residence time. Around 50 μs, this pressure wave combines with the one generated during the voltage drop phase, providing sufficient kinetic energy to eject the ink from the chamber, forming the ink column. Between 60 μs and 150 μs, the ink column gradually undergoes necking due to the influence of surface tension. Finally, based on Plateau–Rayleigh instability, the ink column breaks up, forming droplets. From 160 μs to 260 μs in Figure 6a, the tail of the ink droplet forms unbroken satellite droplets under the combined action of surface tension and viscous forces, which then accelerate towards the main droplet, eventually merging. Figure 6b presents the simulation results of the droplet ejection process. The simulation model successfully reproduces the liquid column formation, break-up, and the formation and acceleration of unbroken satellite droplets towards the main droplet during the ejection process.

In the simulation results, the necking and break-up of the liquid column occur earlier than in the actual experimental results, with a greater degree of necking observed in the simulation. This discrepancy is attributed to the fact that the rheological properties of the ink in the simulation model do not account for the effects of nanometer-sized particles. The high solid content of zirconia nanoparticles in the ink leads to continuous collisions and friction during the ejection process, introducing additional shear forces that increase the viscosity of the ink [44,45]. Additionally, the viscoelasticity of the ink may also be a contributing factor to this phenomenon [40]. This causes the necking rate of the liquid column to decrease, making it harder for the column to contract and break rapidly. Additionally, the interaction forces between the particles further stabilize the liquid column.

**Figure A2:**
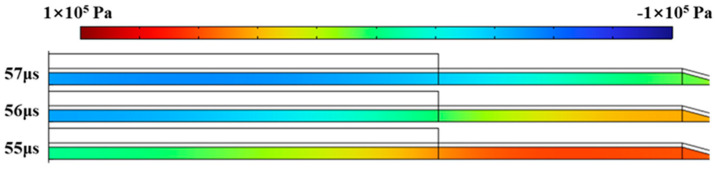
Pressure clouds in the ink chamber at different time points.

**Figure A3:**
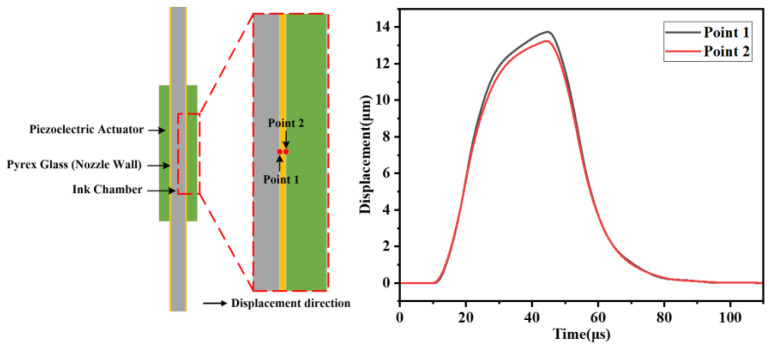
Variation of piezoelectric actuator and Pyrex wall deformation with time.

Figure 6c shows the simulation results of the pressure fluctuation at the nozzle outlet, corresponding to the ejection process shown in Figure 6b. Between 40 μs and 57 μs, the ink at the nozzle outlet gains sufficient kinetic energy under the influence of the positive pressure wave generated by the decreasing driving voltage, causing the ink to start ejecting. From 57 μs to 66 μs, the generation of the negative pressure wave promotes the necking of the liquid column tail, accelerating the droplet formation. During the period from necking to complete droplet break-up, the residual pressure fluctuations at the nozzle outlet deform the tail of the liquid column, leading to the formation of satellite droplets at the droplet tail. Therefore, by optimizing the waveform to suppress the residual pressure fluctuations after ejection, not only can the ejection frequency be increased but the droplet shape can also be effectively improved, reducing the formation of satellite droplets. To provide a more intuitive illustration of the pressure propagation process, Figure A2 in Appendix A.5 presents the pressure distribution within the ink chamber at different time points after the end of the driving voltage. Furthermore, to provide a clearer understanding of the structural dynamics during the jetting process, we have included Figure A3 in Appendix A.5, which presents the temporal deformation of the piezoelectric actuator and the Pyrex wall.

### 3.2. Effect of Waveform Parameters

**Figure 7:**
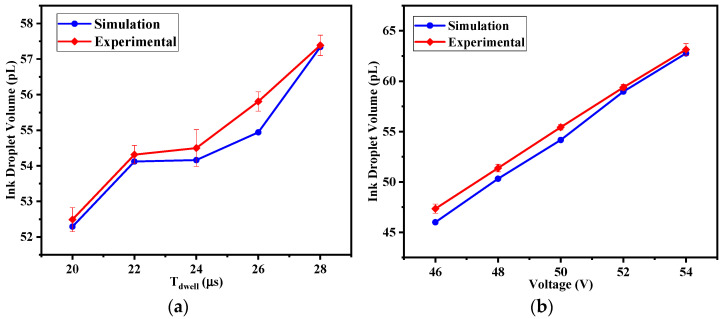
Effect of waveform parameters on droplet volume: (**a**) $T_{dwell}$; (**b**) $V_{1}$.

To further validate the reliability of the simulation model and observe the influence of different single waveform parameters on the ejected droplet, experiments and corresponding simulations were conducted with varying $T_{dwell}$ and $V_{1}$ variables. The resulting droplet volume variation curves are shown in Figure 7a,b. When $V_{1}$ increased from 46 V to 54 V, the pressure fluctuation at the nozzle outlet became more pronounced. In the jetting experiments, the droplet volume increased from 47.35 pL to 63.13 pL, while in the simulation model, the droplet volume increased from 46.00 pL to 62.76 pL, both showing a clear linear growth trend.

During the period when $T_{dwell}$ increased from 20 μs to 28 μs, the nozzle outlet remained under positive pressure wave conditions at the end of the dwell time. Consequently, the positive pressure wave generated by the downward slope of the driving waveform could still overlap with it, causing the droplet volume to show an upward trend in both the experimental and simulation results. In the experiment, the droplet volume increased from 52.49 pL to 57.39 pL, while in the simulation, it increased from 52.29 pL to 57.34 pL. Since the dwell time $T_{dwell}$ primarily affects the propagation and reflection of the pressure waves within the ink chamber, Figure 7a shows a more obvious nonlinear trend. Compared to the voltage amplitude $V_{1}$, the effect of $T_{dwell}$ on droplet volume growth is relatively weak. The experimental and simulation results exhibit consistency in trends, with the maximum deviation being 2.85%. Therefore, the simulation model can be used to replace the experimental process for driven waveform optimization, better quantifying optimization metrics and improving research efficiency.

### 3.3. Desired Pressure Fluctuation

**Figure 8:**
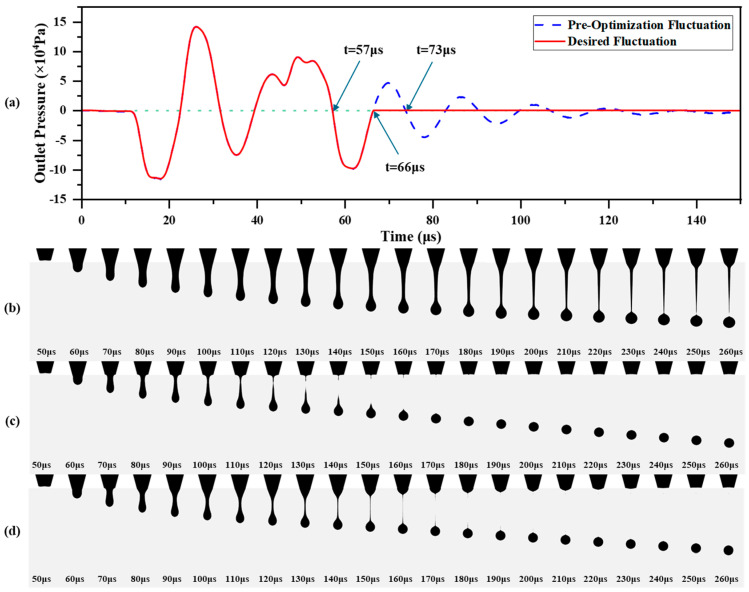
Desired pressure fluctuation and suppression of residual fluctuations at different time points in ejection simulation: (**a**) desired pressure fluctuation; (**b**) 57 µs; (**c**) 66 µs; (**d**) 73 µs.

Based on the single waveform described in Section 2.4 and as shown in Figure 6b,c, the beneficial pressure fluctuation interval at the nozzle outlet during the droplet ejection process is from 0 to 66 μs. By suppressing the pressure fluctuations outside this interval, the desired pressure fluctuation shown in Figure 8a can be obtained. To further validate this, the pressure fluctuation at the time nodes of 57 μs, 66 μs, and 73 μs, where the pressure is zero, was suppressed, and the results were used as input for simulation. The simulation results are shown in Figure 8b–d. Compared to the simulation results shown in Figure 6b, suppressing residual pressure fluctuations clearly eliminates the satellite droplet phenomenon in the droplet ejection process. For the case with the 57 μs time node for suppressing residual pressure fluctuation (Figure 8b), the absence of the negative pressure phase from 57 μs to 66 μs results in a significantly slower necking and break-up speed of the droplet. On the other hand, when the 73 μs time node is used (Figure 8d), although the necking and break-up speed improves, the residual positive pressure fluctuation between 66 μs and 73 μs still causes some ink to be ejected after the tail of the liquid column contracts. Consequently, the droplet break-up time still lags behind the 66 μs time node shown in Figure 8c. By suppressing the pressure fluctuation after the 66 μs time node, as shown in Figure 8c, the desired pressure fluctuation can effectively avoid the satellite droplet phenomenon while also significantly accelerating the droplet formation speed, thus improving the ejection frequency. The desired pressure fluctuation can be achieved by adding an additional single waveform after the first single waveform. The parameters of the additional waveform must be carefully selected and optimized to ensure that the pressure fluctuation at the nozzle outlet under the double waveform drive closely matches the desired pressure fluctuation.

## 4. Waveform Optimization

The droplet ejection process triggered by the driving waveform exhibits significant nonlinear behavior, and traditional experimental optimization methods often lead to local optima and low efficiency. To address this issue, this study will use a CNN regression model combined with a PSO algorithm for waveform optimization. Compared to traditional neural network models, CNNs not only have simpler model parameters but also automatically extract local features and complex hierarchical structures from the training data. PSO, as a heuristic global search algorithm, does not depend on initial parameters and can effectively avoid local optima. In this study, a large amount of droplet ejection process data is obtained from the simulation model and used as the basis for training the CNN regression model. This model establishes a nonlinear mapping relationship between the driving waveform and the pressure fluctuation at the nozzle outlet, specifically the time required for the pressure fluctuation to converge. PSO is then used to further search and optimize the model to obtain the driving waveform that results in the desired pressure fluctuation. Algorithm 1 presents the pseudocode for the waveform optimization method. The parameters of the additional single waveform include the interval time $T_{interval}$, rise time $T_{rise}$, dwell time $T_{dwell}$, and voltage amplitude $V_{2}$, with the fall time set equal to the rise time. The pressure fluctuation convergence time and fitness function are defined as the time $T_{c}\left(T_{interval},T_{rise},T_{dwell},V_{2}\right)$ for the pressure fluctuation at the nozzle outlet to decay to 1.0 × 10^4^ Pa.
**Algorithm 1.** Pseudocode for waveform optimization method—CNN-PSOBegin Input: Ink Droplet Ejection Simulation Data $T_{interval}$$,\;T_{rise}$$,\;T_{dwell}$$,\;V_{2}$$,\;T_{c}$ Initialize the Convolutional Neural Network (CNN) model parameters Train the CNN model while (training stopping condition not met) do   for each batch of data in the training set do       Perform forward propagation to calculate predictions       Compute loss value       Update CNN weights through backpropagation    End End Save the trained CNN model Initialize the particle swarm while (stopping condition not met) do    for each particle in the swarm do      Use the trained CNN model to calculate the fitness value $T_{c}$ of the current particle       if (current fitness value $T_{c}$ is better than the individual best fitness value $T_{c\;best}$) then          Update individual best position $\left(T_{interval\;best},\;T_{rise\;best},\;T_{dwell\;best},\;V_{2\;best}\right)$      End       if (current fitness value $T_{c}$ is better than the global best fitness value $T_{c\;gbest}$) then         Update global best position $\left(T_{interval\;gbest},\;T_{rise\;gbest},\;T_{dwell\;gbest},\;V_{2\;gbest}\right)$      End       Update particle velocity and position   End End End Return the global best position $\left(T_{interval\;gbest},T_{rise\;gbest},T_{dwell\;gbest},V_{2\;gbest}\right)$

### 4.1. Pressure Fluctuation Convergence Regression Model

**Figure 9:**
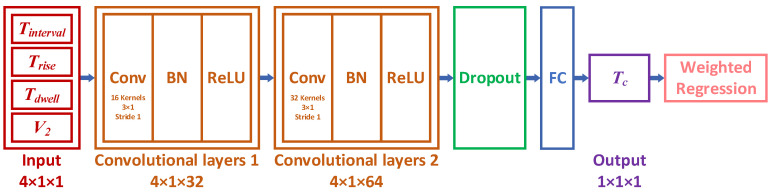
CNN Regression Model Architecture.

This study establishes a CNN regression model, as shown in Figure 9, to predict the pressure fluctuation convergence time under different drive waveform parameters. The waveform parameters are input into the CNN regression model, which then passes through two 2D convolutional layers for feature extraction. The first convolutional layer contains 16 filters, while the second contains 32 filters. Each convolutional layer is followed by a batch normalization layer and a ReLU activation layer to enhance training stability and introduce non-linearity. To preserve more details and improve the model’s prediction performance in small-sample regression, pooling layers are omitted from the CNN. Additionally, a Dropout layer is included to prevent overfitting during training. Since the model will ultimately be used for optimization within the PSO algorithm, with a focus on predicting minimal convergence times, a weighted regression layer replaces the conventional regression layer in the CNN. This change assigns greater weight to the minimal value samples, improving the model’s prediction accuracy for convergence times.

A total of 1575 sets of random drive waveform parameters ($T_{interval}$, $T_{rise}$, $T_{dwell}$, and $V_{2}$) were selected for jetting simulations to obtain the pressure fluctuation convergence time at the nozzle outlet. Moreover, 80% of the data were used as the training set for the CNN regression model, while the remaining 20% were used for testing. During training, the Adam optimizer was applied with a batch size of 32, a maximum of 1200 training iterations, and an initial learning rate of 0.01. A stepwise decay strategy was adopted, reducing the learning rate by 10% every 400 epochs. After training the CNN model, linear regression was used to correct the predicted results.

**Figure 10:**
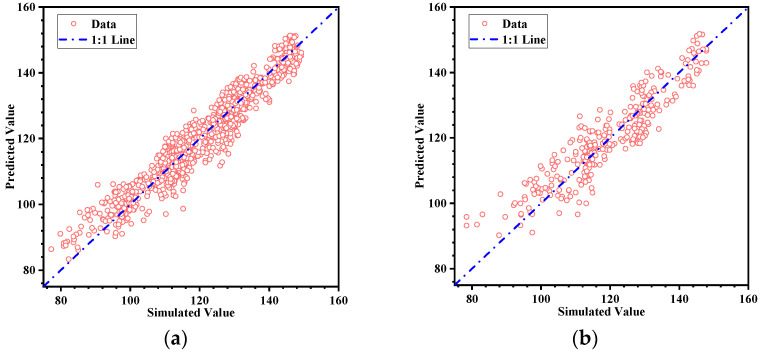
CNN prediction results: (**a**) training set; (**b**) test set.

Figure 10 shows the final prediction accuracy analysis, and Table 1 lists the evaluation metrics for the model. In the training set, the coefficient of determination R^2^ = 0.92012 indicates that the model can explain approximately 92% of the data variance. The mean absolute error (MAE) = 3.1644 and the root mean square error (RMSE) = 4.0159 also show small errors, suggesting a high degree of fit to the training data. For the testing set, although the model’s ability to explain the data decreased slightly, R^2^ still reached 0.84278, indicating a certain degree of generalization ability. Although the MAE and RMSE values are higher for the test set compared to the training set, they remain within a reasonable range. The model demonstrates a balanced performance on both the training and testing sets, with good predictive capability, making it suitable for the optimization of drive waveform parameters in subsequent steps.

### 4.2. Optimization of Drive Waveform Parameters

The nozzle outlet pressure fluctuation convergence time predicted by the CNN regression model is set as the fitness function, and PSO is applied for optimization. During the optimization process, the initial population size is set to 40, and the maximum number of iterations is 100. The driving waveform parameters $T_{interval}$, $T_{rise}$, $T_{dwell}$, and $V_{2}$ are treated as the individual position parameters. The constraints for these parameters are specified according to the desired pressure fluctuation, as shown in Table 2. The inertia weight of PSO is set to 0.8, and both the individual learning factor and the global learning factor are set to 2.

**Figure 11:**
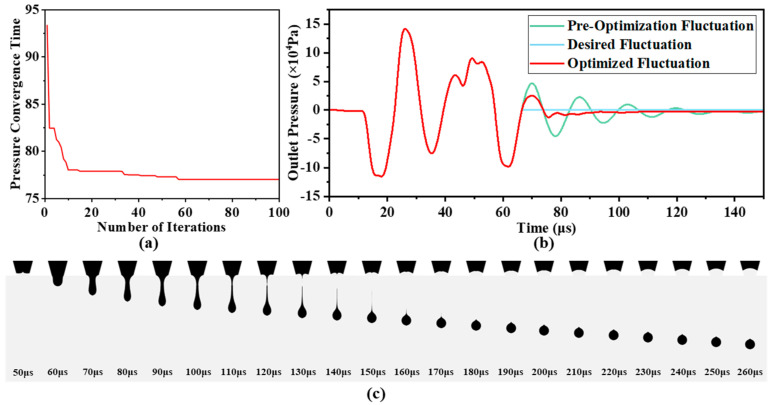
Waveform optimization results: (**a**) PSO iteration process; (**b**) optimized pressure fluctuation at nozzle outlet; (**c**) optimized ink droplet ejection process.

**Figure A4:**
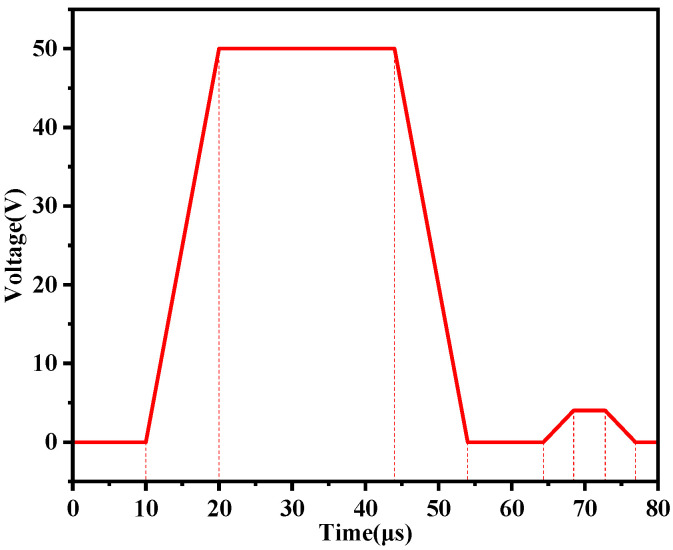
Optimized voltage waveform.

Figure 11a shows the variation curve of the nozzle outlet pressure convergence time during the PSO iteration process. As the number of iterations increases, the pressure convergence time decreases rapidly, reaching the minimum of 77.04 μs after 57 iterations. The corresponding optimized waveform parameters are *T_interval_* = 10.35 μs, *T_rise_* = 4.12 μs, *T_dwell_* = 4.32 μs, and *V*_2_ = 4.02 V. The optimized voltage waveform can be found in Figure A4 of Appendix A.6.

By using the optimized waveform parameters as inputs for simulation, the nozzle outlet pressure fluctuation after optimization is obtained, as shown in Figure 11b, and the inkjet process is shown in Figure 11c. The simulated pressure convergence time is 76.68 μs, with an error of 0.47% compared to the predicted result. Although the desired pressure fluctuation effect was not fully achieved, the optimization significantly suppressed the residual pressure fluctuation after 66 μs, as compared to the pre-optimization waveform. The nozzle outlet pressure convergence time was reduced by approximately 32.19% from the original 113.08 μs. During the inkjet process, no significant satellite droplet formation was observed, and the liquid column necking and break-up time closely matched the desired pressure fluctuation behavior.

## 5. Conclusions

This study focuses on material jetting technology and presents a numerical model for ink droplet jetting that integrates force interactions, fluid dynamics, and multi-physics coupling, including electrical effects. A comparative analysis of the droplet ejection process under single waveform driving reveals a strong correlation between satellite droplet formation and residual pressure fluctuations at the nozzle outlet after the driving waveform ends. Initially, the negative pressure wave accelerates the necking of the liquid column tail, while subsequent pressure fluctuations cause tail deformation and promote satellite droplet formation. By selecting appropriate time nodes to suppress these residual pressure fluctuations, satellite droplets can be effectively avoided, and the liquid column break-up can be accelerated, thus increasing jetting frequency. Further, the study identified a non-linear relationship between the driving waveform and the ink droplet ejection process. Using a CNN regression model, a non-linear mapping was established between the nozzle outlet pressure fluctuation convergence time and driving waveform parameters. The PSO algorithm was then applied to optimize the driving waveform, significantly suppressing the residual pressure fluctuations and bringing the pressure fluctuation closer to the desired waveform. After optimization, the pressure convergence time was reduced by approximately 32.19%, leading to a more stable jetting process with a higher jetting frequency and fewer satellite droplets. While the proposed optimization framework demonstrates robust performance under the Newtonian fluid assumption, additional complexities arising from dynamic surface tension evolution and high-frequency viscoelastic behavior may significantly influence the ligament breakup process. Future studies should incorporate these effects to further refine the predictive accuracy and applicability of the model.

## Figures and Tables

**Table 1:** Evaluation metrics for pressure fluctuation convergence regression model.

Sample	R^2^	MAE	RMSE
Training set	0.92012	3.1644	4.0159
Test set	0.84278	4.6300	5.7168

**Table 2:** PSO position parameter limits.

Parameter	$T_{interval}$	$T_{rise}$	$T_{dwell}$	$V_{2}$
Limit	[7 μs, 17 μs]	[0 μs, 10 μs]	[0 μs, 10 μs]	[0 V, 20 V]

## Data Availability

Data available upon request due to privacy.

## Abbreviations

The following abbreviations are used in this manuscript:

CNN	Convolutional Neural Networks
PSO	Particle Swarm Optimization
DoD	Drop-on-Demand
AI	Artificial Intelligence
3Y-TZP	3 mol% Yttria-Stabilized Tetragonal Zirconia Polycrystals
XRD	X-Ray Diffraction
TEM	Transmission Electron Microscopy
PVA	Polyvinyl alcohol
CCD	Charge Coupled Device

## Appendix A

### Appendix A.1

**Table A1:** Level set parameter.

Parameter	Value
Re-initialization parameter	5 m/s
Interface thickness control parameter	2 μm

### Appendix A.2

**Table A2:** Geometric model dimension parameters.

Structure	Dimensional Parameters
Ink Chamber Inlet Length $l_{1}$(mm)	6.400
Piezoelectric Actuator Length $l_{2}$(mm)	10.400
Ink Chamber Outlet Length $l_{3}$(mm)	6.000
Nozzle Conical Angle θ (°)	75.000
Outer Diameter of Piezoelectric Actuator (mm)	0.648
Outer Diameter of Nozzle Wall (mm)	0.340
Inner Diameter of Nozzle Wall (mm)	0.254
Nozzle Diameter (mm)	0.025

### Appendix A.3

In this section, we have supplemented the relevant data on the mesh. Figure A3 shows the mesh image at the nozzle, while Table A1 presents the maximum peak values of pressure fluctuations generated by the mesh under different characteristic sizes. The mesh characteristic sizes listed in the table correspond to the predefined sizes in COMSOL’s computational fluid dynamics module. From the table, it can be observed that the magnitude of pressure fluctuations is insensitive to the selected mesh sizes, with relative fluctuations being less than 0.05%. Therefore, this study adopts the refined mesh size.

**Table A3:** Maximum peak of pressure fluctuation in mesh generation under different characteristic sizes.

Mesh Feature Size	Maximum Peak of Pressure Fluctuation
Normal mesh	141,480 Pa
Refined mesh	141,510 Pa
Finer mesh	141,520 Pa

### Appendix A.4

This section provides a summary and supplement on the boundary conditions of the droplet ejection model and the selection of the model solver.

All boundary conditions correspond to the physical setup of the experiment and are consistent with the descriptions in Section 2.3.

**Table A4:** Summary of boundary conditions in the COMSOL ejection model.

Region	Physics Domain	Boundary Condition
Nozzle inlet	Fluid (Ink)	Inlet Pressure (−2 kPa)
Nozzle outlet	Fluid (Ink)	Outlet Pressure (0 Pa)
Inner surface of piezoelectric actuator	Solid/Electric	Electric Ground
Outer surface of piezoelectric actuator	Solid/Electric	Prescribed Electric Potential
Outer wall of nozzle at inlet	Solid	Fixed Constraint
Nozzle wall	Multiphase (Level Set)	Wetting Wall with fixed contact angle θ = π/2

To predict the transient jetting behavior under multiphysics coupling, a time-dependent solver was employed in COMSOL 6.2. The solver tolerance was controlled by the physics settings, and the output time steps were specified as a range (0, 1 × 10^−6^, 260 × 10^−6^), corresponding to a simulation time span of 0 to 260 μs with a time step of 1 μs. A segregated solution strategy was adopted to group and iteratively solve the coupled variables, which improves numerical stability. Each sub-step used a direct solver (PARDISO) to ensure robust and efficient computation.
